# Dr. Stockton’s Oration at Portland

**Published:** 1905-08

**Authors:** 


### Dr. Stockton's Oration at Portland.

DR. CHARLES G. STOCKTON, of this city, professor of medicine at the University of Buffalo, delivered the oration on medicine at the recent meeting of the American Medical Association at Portland, Oregon. Elsewhere in this issue we publish the text of his discourse which we feel sure will interest everyone of our readers, and especially those who have been Dr. Stockton's associates and pupils at the university.

When our distinguished colleague was appointed to this office a year ago, we predicted that his oration would be an important feature of the meeting at Portland, and without doubt the unanimous verdict, not only of those who heard his effort but also of that larger audience which reads it in the journals, will be an approval of our prediction. But the oration was more than a feature, it was an event of moment. The subject chosen was not only exceptional but it was handled in a most skilful and entertaining manner, while its rhetoric was faultless. It established a precedent out of the usual beaten track for orators on similar occasions, and sets an example that will prove valuable to those who in future are designated to perform similar functions.

The material of the oration affords food for serious thought. It will awaken an interest in the aged, stimulate healthy professional efforts to prevent presenility, while adherence to its tenets will serve to render old age a cheerful and comforting condition, and to relieve it of the sadness and distress that too often attend it.

---

THE *New York Medical Journal* has administered a merited rebuke to the *California State Journal of Medicine* for a libel which the latter published concerning the former. The editor of the California *Journal* has made an abject apology for his discourteous article and we presume this will end the matter.

In its issue of July 1, the *New York Medical Journal* publishes the correspondence in full, together with editorial comments, under the heading "A Warning to Calumniators." The action of our esteemed contemporary merits and should receive the approbation of every medical journal in the country. Some have already commented upon the incident and others no doubt will do so. The Saint Louis *Medical Review,* July 8, in an extensive commentary says in conclusion: "The *California State Journal of Medicine* is a little given, as a continuous performance, to the search for motes in the eyes of others." Perhaps the sophomoric editor will now close up his "continuous performance" show in view of the castigation he has received.

---

THE JOURNAL with this issue enters upon the sixty-first year of its publication. It accentuates the fact by changing the tint of its cover, and by dropping the double series enumeration heretofore in vogue. Its sixty-first volume will be so labeled, and subscribers and others who bind it can easily renumber the previous volumes to make them read in consecutive order.

